# Breeding More Crops in Less Time: A Perspective on Speed Breeding

**DOI:** 10.3390/biology11020275

**Published:** 2022-02-10

**Authors:** Kajal Samantara, Abhishek Bohra, Sourav Ranjan Mohapatra, Riry Prihatini, Flora Asibe, Lokendra Singh, Vincent P. Reyes, Abha Tiwari, Alok Kumar Maurya, Janine S. Croser, Shabir Hussain Wani, Kadambot H. M. Siddique, Rajeev K. Varshney

**Affiliations:** 1 Department of Genetics and Plant Breeding, Centurion University of Technology and Management, Parlakhemundi 761211, Odisha, India; kajalsam27@gmail.com; 2 ICAR-Indian Institute of Pulses Research (IIPR), Kanpur 208024, Uttar Pradesh, India; abhi.omics@gmail.com (A.B.); aabha1tewarii@gmail.com (A.T.); alokmaurya994@gmail.com (A.K.M.); 3 Division of Genetics and Tree Improvement, Forest Research Institute, Dehradun 173230, Uttarakhand, India; mohapatrasourav23@gmail.com; 4 Indonesian Tropical Fruit Research Institute, Solok 27301, West Sumatera, Indonesia; riry.prihatini@gmail.com; 5 International Institute of Tropical Agriculture, Ibadan 200001, Oyo State, Nigeria; f.asibe@cgiar.org; 6 Department of Genetics and Plant Breeding, Agriculture and Forestry University, Chitwan 44200, Nepal; lokendrasingh2053@gmail.com; 7 Graduate School of Bioagricultural Sciences, Nagoya University, Chikusa, Nagoya 464-8601, Aichi, Japan; reyes.vincent.pamugas.r9@f.mail.nagoya-u.ac.jp; 8 The UWA Institute of Agriculture, The University of Western Australia, Perth, WA 6009, Australia; janine.croser@uwa.edu.au; 9 Mountain Research Center for Field Crops, Sher-e-Kashmir University of Agricultural Sciences and Technology of Kashmir, Anantnag Khudwani, Srinagar 192101, Jammu and Kashmir, India; 10 Centre of Excellence in Genomics and Systems Biology, International Crops Research Institute for the Semi-Arid Tropics (ICRISAT), Hyderabad 502324, Andhra Pradesh, India; 11 State Agricultural Biotechnology Centre, Centre for Crop and Food Innovation, Murdoch University, Murdoch, WA 6150, Australia

**Keywords:** breeding cycle, genetic gain, genomic selection, gene editing, photoperiod, single seed descent

## Abstract

**Simple Summary:**

Feeding our growing population is one of the primary concerns of plant breeders. Plant breeding needs to deliver a steady stream of modern cultivars in a time- and resource-efficient manner. This review discusses the speed breeding (SB) techniques which allow breeders to advance the crop generation in a shorter period of time. In addition, we highlight the current SB applications in major crops and explore ways to integrate SB with new breeding techniques for efficient and faster production of stable lines for basic and applied research.

**Abstract:**

Breeding crops in a conventional way demands considerable time, space, inputs for selection, and the subsequent crossing of desirable plants. The duration of the seed-to-seed cycle is one of the crucial bottlenecks in the progress of plant research and breeding. In this context, speed breeding (SB), relying mainly on photoperiod extension, temperature control, and early seed harvest, has the potential to accelerate the rate of plant improvement. Well demonstrated in the case of long-day plants, the SB protocols are being extended to short-day plants to reduce the generation interval time. Flexibility in SB protocols allows them to align and integrate with diverse research purposes including population development, genomic selection, phenotyping, and genomic editing. In this review, we discuss the different SB methodologies and their application to hasten future plant improvement. Though SB has been extensively used in plant phenotyping and the pyramiding of multiple traits for the development of new crop varieties, certain challenges and limitations hamper its widespread application across diverse crops. However, the existing constraints can be resolved by further optimization of the SB protocols for critical food crops and their efficient integration in plant breeding pipelines.

## 1. Introduction

The current human population is around 7.8 billion and is estimated to reach nearly 9.9 billion by 2050 [1]. Climatic fluctuations involving rising temperatures, more frequent floods, and drought are predicted to lead to novel diseases and more frequent pest outbreaks, requiring an agile plant breeding response [2]. Lin et al. [3] highlighted the urgent need for increasing the current rate of genetic gain of critical food crops to safeguard global food security. Improving the rate of genetic gain will rely on accelerated crop breeding pipelines in order to allow rapid delivery of improved crop varieties. As inferred by the breeder’s equation [4], plant breeding can be accelerated by improving factors that influence the genetic gain per unit time [5,6,7], crucially, the breeding cycle time (t) [8].

**Figure 1:**
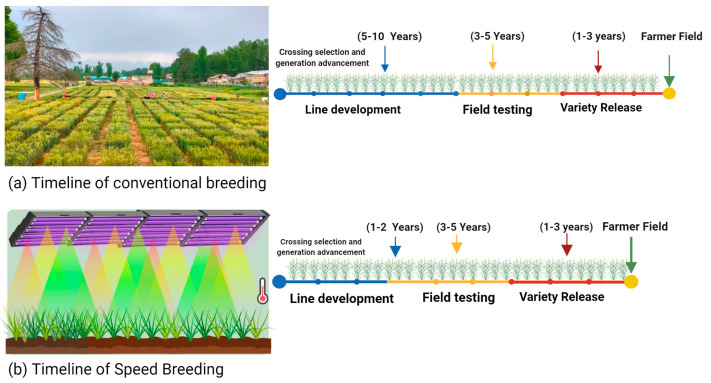
Timelines of varietal development with (**a**) conventional breeding and (**b**) speed breeding. The image was created using BioRender (https://biorender.com/ accessed on 20 January 2022).

Since the 1940s, the speed of plant lifecycle turnover has been manipulated in plant breeding using techniques such as single-seed descent [9,10] and shuttle breeding [11]. More recently, researchers have manipulated controlled-environment (CE) growth conditions to further truncate plant lifecycle time. Techniques to enhance the cycle turnover are widely grouped under the term speed breeding (SB) (Figure 1) [12] and include accelerated single-seed descent (aSSD: rapid development of homozygous lines), rapid generation cycling (RGC: more breeding cycles per year using DNA marker technology), fast generation cycling (FGC: more generations per year using stressed conditions and in vitro culture of immature embryos), and rapid generation turnover (RGT: increasing number of generations per year using immature seed harvest and photoperiod response). Since the early 21st century, this suite of SB techniques has been applied across economically and scientifically important model, crop, and pasture families, including Poaceae, Fabaceae, and Brassicaceae, to achieve up to three-fold improvement in annual generation turnover compared to conventional generation advancement systems (Table 1).

Speed breeding techniques can be used to expedite breeding outcomes including the generation of crosses, mapping populations, and evaluation of agronomic traits of interest. Plants are grown under CE conditions, and researchers manipulate day/night temperature, available light spectrum and intensity, and photoperiod duration in order to reduce time to floral initiation and hasten embryo development and seed maturity [13,14,15,16,17]. Particular emphasis is placed on light, with plants responding to changes in light duration and quality by compressing their time to flowering. The use of artificial electric lamps for accelerating plant growth and development has been long established [18,19]. Photoperiod extension has since been used widely in long-day species to manipulate time to flowering [20]. The advent of advanced LED lighting systems complemented efforts to accelerate lifecycle turnover, enabling manipulation of wavelength composition to trigger light responses, such as shade avoidance, and encourage rapid progression to flowering [21,22,23,24,25].

**Table 1:** List of crops where speed breeding has increased generation turnover.

Type of Photoperiod	Family	Species	Generations/Year	Reference
Long day	Poaceae	Oat (*Avena sativa*)	~7 generations	Liu et al. [26]
		Barley (*Hordeum vulgare*)	~6 generations	Hickey et al. [15]
		Wheat (*Triticum aestivum*)	4–6 generations	Mukade et al. [27]
	Fabaceae	Clover (*Trifolium subterraneum*)	2.7–6.1 generations	Pazos-Navarro et al. [21]
		Lentil (*Lens culinaris*)	~8 generations	Mobini et al. [28]
		Chickpea (*Cicer arietinum*)	~6 generations	Watson et al. [29]; Atieno et al. [30]
		Pea (*Pisum sativum*)	6.8 generations	Ochatt et al. [31]; Ribalta et al. [22]
			5 generations	Mobini and Warkentin [32]
		Faba bean (*Vicia faba*)	7 generations	Mobini et al. [28]
		Narrow-leaf lupin (*Lupinus angustifolius*)	5 generations	Croser et al. [14]
	Brassicaceae	Rapeseed (*Brassica napus*)	~5 generations	Watson et al. [29]
	Linaceae	Flax (*Linum usitatissimum*)	~3 generations	Sysoeva et al. [20]
Short day	Poaceae	Rice (*Oryza sativa*)	~4–5 generations	Rana et al. [33]; Collard et al. [34]
		Sorghum (*Sorghum bicolor*)	4 generations	Forster et al. [35]
	Fabaceae	Soybean (*Glycine max*)	~5 generations	Nagatoshi and Fujita [36]; Jahne et al. [17]
		Pigeonpea (*Cajanus cajan*)	~4 generations	Saxena et al. [37]
		Bambara groundnut (*Vigna subterranea* )	~4 generations	Ochatt et al. [31]
		Groundnut (*Arachis hypogaea*)	~4 generations	O’Connor et al. [38]
	Amaranthaceae	Grain amaranthus (*Amaranthus* spp.)	~6 generations	Stetter et al. [39]

The availability of a low-cost growth room design highlights the versatility of the SB ‘recipe’, which can be tailored according to the local resources and purposes [29]. SB technology has facilitated rapid phenotyping in wheat and the analysis of multiple disease-resistance traits in European two-rowed barley [15,30]. A combination of SB technology and marker-assisted selection (MAS) has accelerated development of herbicide-tolerant chickpea [40] and the introgression of valuable allelic variation from wild relatives in lentil [41]. These practical breeding outcomes highlight the potential of the global suite of SB techniques to substantially accelerate genetic gain.

## 2. Flexible SB Systems for Fast-Tracking Applied and Basic Research

Early SB activities relied on in vivo–in vitro cycling or full in vitro lifecycle turnover [7,14]. However, it is the fully in vivo systems that have been most widely applied in improvement programs. Watson et al. [29] presented three different SB facilities, customizable according to the resource availability. SB I consisted of CE plant growth chambers with a photoperiod of 22 h provided by white LED bulbs, far-red bulbs, and ceramic metal hydrargyrum quartz iodide bulbs and 22 °C day/17 °C night temperature. When grown under these conditions, wheat (*Triticum aestivum*, *T. durum*), barley (*Hordeum vulgare*), and purple false brome (*Brachypodium distachyon*) flowered in half the time of controls grown in unregulated glasshouse conditions during spring and early summer. Germination rates and seed viability remained unaffected by the accelerated growth conditions, validating the potential of this technology for rapid crop improvement. A slightly modified setup, referred to as SB II, employed the same temperature conditions as SB I with a 22 h photoperiod provided by high-pressure sodium vapor lamps. Harvesting of immature seeds and cold treatment were additionally performed under SB II to further reduce generation time. Results from the growth stage for wheat, barley, canola, and chickpea plants demonstrated accelerated plant development and uniformity in time to anthesis under SB conditions compared to the control genotypes grown under identical glasshouse settings without supplemental lights. Under SB II conditions, wheat plants produced significantly more spikes, maintained grain number, and had early maturity within 14 days after flowering. An alternative, cost-effective SB III system included a 3 m^3^ insulated room, seven LB-8 LED light crates, and a 1.5 HP inverter split system domestic air conditioner. The lighting was adjusted to a 12 h photoperiod for four weeks followed by 18 h. The temperatures were maintained at 18 °C during dark and 21 °C during light. Temperature control systems should be carefully considered in SB systems to influence the rate of plant development. Several generations can be achieved in a plant breeding program in a short time by elevating temperature, and SB techniques, in general, adopt late-spring-type temperatures. Likewise, 60–70% humidity is recommended for optimum plant growth and accelerated breeding. The combination of photoperiod, temperature, and humidity in a greenhouse chamber increases the rate of plant development in comparison to the field conditions or conventional glasshouse conditions [22,25].

## 3. SB Applications in Research and Breeding

Applications of SB include the development of biparental and more complex mapping populations, pyramiding traits, hastening backcrosses, phenotyping adult plant traits, mutant studies, and genetic transformation experiments [7,29]. Recent research has shown the power of combining emerging techniques, such as gene editing, high-throughput phenotyping and genotyping, genomic selection (GS), and MAS, with SB for accelerating crop improvement [30,40,42,43,44,45,46]. Furthermore, the cost and space requirements for producing a large number of inbred lines can be minimized by planting them at high plant densities [47]. SB helps overcome challenges associated with double haploid (DH) technology such as low germination rate, poor vigor, and sometimes distorted growth [48]. For genetic mapping purposes, recombinant inbred lines (RILs) developed after multiple generations of self-fertilization can be advantageous over DH due to the multiple meiotic events that occur during the repeated fertilization and the resulting higher recombination frequency. Similarly, advancement and evaluation of segregating generations can be done with SSD in a short period under SB conditions [6], which is time saving and cost efficient compared to the conventional pedigree breeding method [17]. This technique was effective for shortening the generation period, resulting in a three times higher generation turnover than shuttle breeding [49].

## 4. Model Species

In *Arabidopsis thaliana*, seed germination on medium incorporating phytohormone benzylaminopurine and plant auxin-mimic picloram led to seed set approximately 40 to 45 days after sowing. The second-generation seeds were sown on half-strength, hormone-free MS medium resulting in accelerated time to flowering and fruiting compared to the first generation. The length of the second and subsequent cycles could, thus, be cut in half compared to the first generation, allowing for up to 13 generations per year [10]. For pea, Ribalta et al. [22] standardized a protocol to achieve up to six generations per year and identified markers for ascertaining physiological maturity of embryos. Immature seeds, in which embryos had reached physiological maturity, could be reliably harvested and germinated without in vitro intervention. Further study identified a shift in the expression pattern of hormones related to embryo development, with compression of the development when grown under accelerated SSD, CE conditions. In woody, perennial plants, environmental conditions affect the length of the juvenile phase. In apple, field-grown seedlings typically require 5 years to flower, but growth can be accelerated to the adult reproductive phase in a comparatively short time such as 10 months [50]. However, such approaches face challenges at later stages when managing big plants under CE becomes very difficult. The dormancy of temperate-zone trees can also be overcome by maintaining low temperature under high moisture conditions [51], providing a way to hasten the breeding cycle.

### 4.1. Cereals

Researchers have explored novel approaches to reduce the time required to obtain homozygous lines after hybridization to expeditiously breed cereal varieties. For example, four to six generations of wheat were obtained following the harvesting of immature seeds after 15–20 days of anthesis and the treating of the seeds with H_2_O_2_ at a low temperature [27]. A later study by De Pauw and Clarke [52] improved the germination response of wheat seeds by extending the duration of H_2_O_2_ treatment at a low temperature (11 °C) and, depending upon the cultivar, the generation time was reduced by 12–23 days. Similarly, Robertson and Curtis [53] also observed more than 90% germination for air-dried seeds harvested after 21 days of anthesis. In rice, Japanese researchers used generation advancement methods to breed ‘Nipponbare’ and several other cultivars [54]. A breakthrough in the generation acceleration procedure of rice was the development of the biotron breeding system (BBS) that accelerates the breeding cycle by regulating temperature, photoperiod, and CO2 level, in combination with embryo rescue and tiller removal [55]. The utility of the BBS was evidenced by the marked reduction in the generation intervals (two months) of ‘Nipponbare’. Tanaka et al. [54] reduced the generation intervals of ‘Nipponbare’ by three months using the BBS without embryo rescue and tiller removal, thus, enhancing the feasibility of the BBS adoption for rapid generation advancement. More recently, researchers obtained four to five generations of rice per year [12].

The SB technique has been used efficiently in wheat for the rapid screening of multiple traits related to diseases, such as leaf rust, and root architecture and for evaluating plant height and flowering time [12,56]. SB has been implicated for screening drought-tolerance traits in barley [12,57]. A modified, backcrossing methodology, in combination with SB, was used for two years in the development of resistant lines of barley that were otherwise susceptible to different diseases including rust and spot blotch [15]. Similarly, the embryo rescue method and direct germination of immature seeds can be applied in sorghum to significantly reduce the time required for the breeding cycle [58]. Increasing the photoperiod and a foliar mineral supplement are also shown to reduce time to anthesis for a higher generation turnover in oats [54].

### 4.2. Oilseeds

The possibility of viable seed production through precocious germination was shown in soybean [59]. Later, Roumet and Morin [60] demonstrated a growth cycle truncated from 130–140 to 65–70 days using precocious germination of immature, pre-treated pods. Nagatoshi and Fujita [36] developed a standardized rapid generation advancement protocol for high-quality, Japanese, soybean cultivar Enrej, which reduced crop duration from 102–132 days to 70 days. The availability of such methods enables five generations per year instead of one to two generations in a year. In the same way, Watson et al. [29] optimized an SB protocol in canola to enhance the generation turnover and facilitate phenotyping of the pod-shattering trait. For this, five canola cultivars susceptible to pod shattering were grown in environment-controlled growth chambers. Using the embryo rescue technique, Dagustu et al. [61] established a short breeding period protocol for sunflower that can be used to shorten the generation time in a breeding program. For this, seed embryos were cultured in MS media with 2% sucrose and 0.8% agar at pH 5.6–5.7 after 10–12 days of pollination, as previously used in tobacco [62].

### 4.3. Legumes

An in vitro-assisted SSD technique in clover (*Trifolium subterraneum* L.) accelerates the generation cycle by minimizing time to flowering by growth under regulated temperature, along with an expanded photoperiod, truncated seed-filling period, and embryo rescue. Growing immature seed helps overcoming the problem of seed dormancy. This technique enables 2.7–6.1 generations per year over a wide range of clover genotypes [21].

Generation acceleration protocols have been optimized in many legume species, especially temperate pulses that positively respond to photoperiod extension owing to their facultative, long-day nature [14]. For example, continuous light in conjunction with optimal temperature and humidity in a greenhouse facility led to an increased rate of plant growth in peanut [17]. Compared to greenhouse conditions, the in vitro protocols, in combination with in vivo manipulation, worked better for shortening generation cycles in pea and bambara groundnut, whereas an in vivo-only strategy showed promising results in the case of peas and grass peas [7]. Similarly, Espósito et al. [63] devised an in vitro-only strategy to generate an adequate number of F_2_ plants in pea breeding programs. Lentil can be grown in vitro using a tissue culture method with agar as the substrate and MS as the medium or a hydroponic system with perlite as the substrate and HS as the medium or agar as the substrate and Hestrin–Schramm (HS) as the medium, which enables eight generations of lentil within one year [28]. This study expanded the in vitro rapid generation technology of lentil to faba bean and evaluated the effect of the hydroponic method, tissue culture, and intermediate method on accelerating anthesis time and seed set rates. The generation time was 54 days, including 18 days for immature seeds to be ready for embryo rescue, resulting in 6.8 generations each year as opposed to one in the field and three in the greenhouse. A more recent study in chickpea (*Cicer arietinum* L.) reduced seed-to-seed cycle time based on induction of early flowering and germination of immature seeds [64]. In pigeonpea, a rapid generation advancement strategy demonstrating 100% germination from immature seeds harvested from 35-day-old plants provided novel avenues for growing three to four generations in a year [37]. Considerations of light quality allowed researchers to extend these SB protocols to soybean, a short-day legume crop. A 10 h photoperiod using a blue-light-enriched, far-red-deprived light spectrum enabled plants to mature within 77 days after sowing, thus, allowing five generations of soybean in a year [17]. A recent study in groundnut combined the single-seed chipping (SSC) process with high-throughput genotyping (HTPG) and SB. A small portion of cotyledon from the posterior end of the seed was used for DNA extraction. A germination rate of 95–99% was observed from the chipped seeds. The study led to a time saving of 6–8 months following the implementation of this integrated approach in groundnut research and breeding [65]. Since 2016, SB has been integrated within all cool-season legume public breeding programs in Australia, with more than 45,000 individuals processed through an aSSD platform at the University of Western Australia. The resulting RILs have been used for gene-trait associations [43,44,45,46], and SB has been integrated with other technologies to accelerate cultivar development pipelines [40].

The growing numbers of examples of SB in long-day and more recently in short-day plants attest to its broad utility in breeding programs, allowing faster homozygosity, the creation of mapping populations, and a significant reduction in time, space, and resources for cultivar development.

### 4.4. Fruit Crops

Many fruit crops undergo a long juvenile phase before flowering, in some cases, taking >20 years [51]. SB techniques have led to vigorous vegetative growth and early flowering in apple (ten months instead of five years) and chestnut (two years instead of seven years) [51,66]. The development of a new cultivar with desirable traits was achieved in apple using SB technology, which is based on transgenic, early-flowering plants and MAS [67]. Several of the clonally propagated crops, such as banana, roots, and tubers (not fruit crops), have begun to utilize SB in order to reduce flowering time and increase flowering rate, as well as the predictability of flowering, for the introduction of disease-resistance traits, as exemplified by bacterial wilt in banana [68,69].

### 4.5. Vegetable Crops

Extending the photoperiod has shortened generation intervals in vegetables, such as pepper, tomato, and amaranth, which respond effectively to increased daylight [39,70]. In tomato, germination of immature seeds from different maturity levels provided new possibilities to achieve five generations instead of the conventionally grown three [71]. Similarly, in pepper and tomato, in vitro germination of immature embryos enabled authors to obtain one more generation compared to conventional breeding practice [72,73]. In grain amaranth, photoperiod manipulation was reported to be helpful in flowering synchronization in different germplasm lines, which, in combination with DNA marker technology, led to the development and identification of true hybrids, thus, accelerating the breeding program [39]. Other methodologies that can improve generation turnover in vegetables by promoting early flowering involve higher expression of flowering genes such as the *CaFT-LIKE* gene in pepper [74]. Similarly, as demonstrated by Velez-Ramirez et al. [75], in tomato, introgression of the gene *CAB-13* can impart tolerance to continuous light, thus, adapting plants to extended photoperiods.

## 5. Opportunities for Combining SB with Modern Breeding and Phenotyping Tools

**Figure 2:**
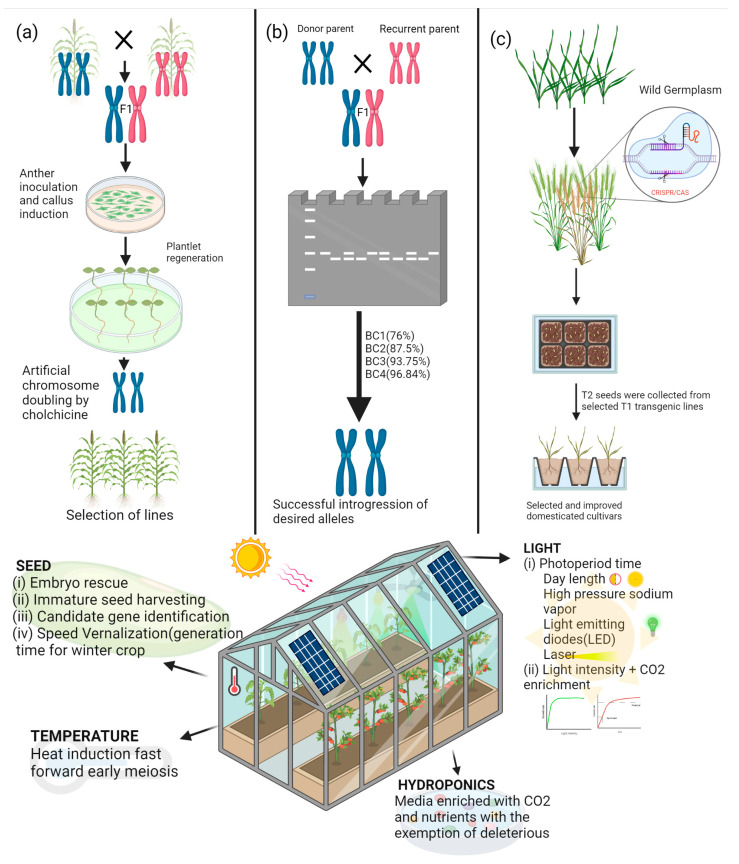
Integration of SB with other breeding techniques accelerates the rate of progress. The homozygous lines for research and breeding purposes can be obtained expeditiously through hastening procedures aimed at (**a**) DH production, (**b**) marker-assisted selection, and (**c**) gene editing. The image was created using BioRender (https://biorender.com/ accessed on 20 January 2022).

In the 21st century, crop improvement was revolutionized by DNA marker technology and genomics-assisted breeding. More recently, genome editing techniques, based on site-specific nucleases, are being applied to improve agricultural procedures by developing superior plant varieties. The current genome editing protocols can be enhanced by integration with SB as the edited plants can be grown under SB conditions to rapidly attain edited seeds, thereby accelerating homozygosity and the potential rate of genetic gain. Given this, genome editing using a CRISPR/Cas9 system and SB will likely become popular as the technology is adapted to new species. Desirable lines produced from genome editing can be preselected at the T_1_ generation with strict evaluation conducted at the T_2_ generation for elimination of off-target genotypes (Figure 2). The application of SB in combination with genome editing has been demonstrated in *Brassica napus*, *B. oleracea*, and soybean [76,77,78].

Integration of SB into MAS/marker-assisted backcrossing (MABC) can also serve as a platform for the introgression of beneficial alleles in various crops. MAS/MABC is an established method for improving yield, biotic stress, and abiotic stress among major crops [7]. For example, *pi21* is known to confer quantitative resistance to rice blast. This gene has been successfully introgressed in select rice cultivars [79]. The DNA-marker-assisted approaches help minimize the problem of linkage drag, i.e., unintended introgression of unfavorable alleles with target loci. Backcrossing procedures demand considerable time for improvement of the recipient genotypes. Through the integration of SB, the progress of backcrossing or MABC can further be accelerated for quick transfer of the target trait(s).

SB has been applied for evaluating various stages in plant breeding programs. Genomic selection (GS) was combined with SB in spring wheat to increase genetic gain vis-à-vis complex traits [80]. SB was used in the phenotyping of specific traits in the training population of wheat, selection candidate development, and selection candidate phenotyping steps. Concerning the indirect selection in targeted-population SB environments, it was concluded that plant height and flowering time can be predicted with accuracy comparable to direct field selection. Speed breeding also facilitates a higher rate of genetic gain compared to direct field phenotyping [81]. Multi-trait phenotyping protocols have been optimized for evaluating crown root resistance and leaf rust tolerance in wheat under a SB system. The effectiveness of the early-generation selection of F_2_ population for multiple traits was tested in order to estimate phenotypic response. The method illustrates efficient exploitation of resources by analyzing multiple traits for the same group of plants. Selection in the early generation under SB improves genetic gain in breeding programs, as well as curtailing the time required to incorporate desirable traits in breeding populations [40,49]. Phenotyping of wheat or other important crops under SB conditions can be further improved through MAS.

High-throughput phenotyping is among the key breakthroughs of 21st-century agricultural research that have substantially overcome long-standing obstacles in the progress of plant breeding. Conducting high-throughput phenotyping under SB conditions creates novel avenues for discovery and incorporation of beneficial traits in a resource-efficient manner [82]. For example, targeting proxy traits, such as seminal root number and angle of wheat seedlings, under SB conditions (higher planting density, temperature control, and extended photoperiod) facilitated rapid selection for improved root architecture of mature plants [83]. Similarly, imaging technology permitted collection of field plot images at 7400 plots/h based on color traits in wheat [84]. The technology using non-human-operated aerial vehicles showed strong correlation with increased grain yield compared to terrestrial sensing.

For the breeding of salt-tolerant rice, Rana et al. [33] applied SB to rapidly derive a BC3F3 population from highly tolerant and high-yield parents. The population was assayed with SNP markers and provided promising salt-tolerant variety candidates [33]. Plants grown under SB demonstrated significant differences from the control plants in terms of various yield-predicting component traits, viz., panicle length, tiller number, spikelet number per panicle, and normalized difference vegetation index during panicle initiation until mid-grain panicle filling [85]. Hence, these studies can be considered as a means to gain more extensive knowledge on plant growth and developmental processes for further crop trait improvement studies.

The single-seed descent (SSD) method under SB conditions enables the generation of near-homozygous lines in a single year and provides a greater scope for preserving genetic diversity in a crop breeding program. Likewise, rapid development of recombinant inbred lines (RILs) using SB technology has given impetus to studies aiming at identification of gene-trait associations for breeding applications [22,43,44,45,46]. Again, DNA marker technology in combination with SB helps design new strategies for basic and applied research; for instance, SB can allow the quick development of new generation mapping populations derived from multi-parental populations such as MAGIC and NAM [86,87].

## 6. Challenges and Limitations

**Figure 3:**
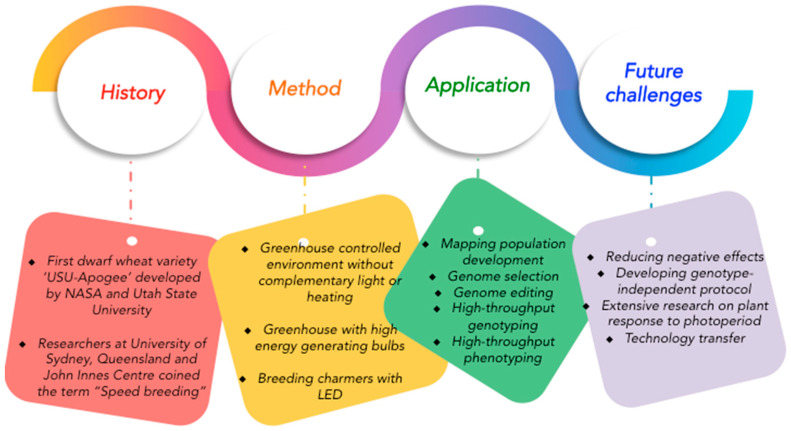
Retrospect, current methods, applications, and challenges of speed breeding.

As described above, SB is a powerful tool for accelerating the rate of genetic gain in different plant species; however, it has limitations (Figure 3). A key limitation is access to CE conditions suited to the rapid cycling of the target species. SB settings become expensive if sophisticated CE facilities are not readily available and combining SB with other techniques, such as embryo rescue and MAS, requires additional resources and expertise. Other challenges include continuous supply of electricity and temperature maintenance, for example, during winter [38]. While not so problematic in developed countries, routine use of SB for research and breeding remains a challenge in resource-poor countries due to limited infrastructures, poor expertise, and limited collaborations with international organizations [88].

Once SB is established, species can exhibit genotypic differences in response to intensive growth conditions [54,85]. The intensive growth conditions often result in limited seed yield, which can constrain subsequent field evaluations [89]. The use of next-generation sparse phenotyping field trial designs can assist in overcoming low seed numbers [29]. An excessive photoperiod can limit plant growth and may be correlated to photo-oxidation, high starch production, and elevated levels of stress hormones [16,90]. Similarly, the harvesting of immature seed may interfere with the phenotyping of seed traits. Importantly, in pushing for speed, plants are grown at the edge of their physiological capability, and conditions conducive to fast cycle turnover are often detrimental to the plants ability to defend itself and, without careful management, can lead to catastrophic losses of valuable breeding material. Mitigation comes from adaptation of conditions to determine photoperiod saturation and temperature limits for each species and, in some cases, for genotypes within species. Maintaining back-up seed from each individual through each generation also provides insurance against genetic loss in the event of one generation being affected by disease, pests, or power loss in CE or similar.

Phenotyping for agronomic traits can be undertaken in conjunction with SB; however, care is required as phenotypic expression can be biased under CE conditions. As a result, phenotyping of field crops under SB should be validated in the field to certify that trait expression is associated to the field environment. For example, boron tolerance was reliability discriminated in pea grown hydroponically to integrate with SB conditions, and, in wheat, SB techniques were combined with phenotype screening such as disease resistance [81,91]. Several agronomic characters, namely flowering time and plant height, when recorded in SB conditions, are related to field-based determination and production, thus, can facilitate indirect selection [81]. Nevertheless, several characters may not be consistently phenotyped due to the cross-over interaction between genotypes and growth systems, as observed in the case of plant height and flowering time in oat [89]. Other major challenges while growing plants in SB conditions include pest and disease management under such intensive conditions and the need for tracking of individuals when developing mapping populations for gene-discovery purposes.

## 7. Conclusions

The plant research community has yet to achieve the scale and speed of plant improvement required to effectively feed a burgeoning world population in the face of a changing climate. The coupling of emerging genomics techniques with methods for rapid gene fixation offers the potential to transform the rate of genetic gain in breeding programs [6,90]. Since its introduction, SB has accelerated the breeding programs of many economically important species. Relying on light and temperature manipulation, along with physical containment, SB impacts different phases of plant breeding by hastening the breeding cycle. SB enables rapid progression to homozygosity and evaluation of already developed or transformed lines, viz., gene-edited crops and transgenic crops. The SB techniques also facilitate resource-efficient genotyping and phenotyping; however, further research is required to assess and mitigate the negative effects of SB conditions on plant growth and development. The SB protocols are now available for small- or broad-scale adoption and further modifications based on local needs/innovations. The SB protocols can, thus, be progressively improved and combined with modern breeding techniques to realize their potential for the identification and transfer of genes critical to crop resilience and adaptation [92]. Collaborative international partnerships involving multi-disciplinary teams are needed to encourage the integration of SB systems in basic and applied research, particularly in developing countries.

## Data Availability

Not applicable.

## References

[1] IISD World Population to Reach 9.9 Billion by 2050. https://sdg.iisd.org/news/world-population-to-reach-9-9-billion-by-2050/.

[2] Hussain B. (2015). Modernization in plant breeding approaches for improving biotic stress resistance in crop plants. Turk. J. Agric. For..

[3] Lin Z., Cogan N.O.I., Pembleton L.W., Spangenberg G.C., Forster J.W., Hayes B.J., Daetwyler H.D. (2016). Genetic Gain and Inbreeding from Genomic Selection in a Simulated Commercial Breeding Program for Perennial Ryegrass. Plant Genome..

[4] Moose S.P., Mumm R.H. (2008). Molecular plant breeding as the foundation for 21st century crop improvement. Plant Physiol..

[5] Bohra A., Jha U.C., Godwin I.D., Varshney R.K. (2020). Genomic interventions for sustainable agriculture. Plant Biotechnol. J..

[6] Sinha P., Singh V.K., Bohra A., Kumar A., Reif J.C., Varshney R.K. (2021). Genomics and breeding innovations for enhancing genetic gain for climate resilience and nutrition traits. Theor. Appl. Genet..

[7] Varshney R.K., Bohra A., Yu J., Graner A., Zhang Q., Sorrells M.E. (2021). Designing Future Crops: Genomics-Assisted Breeding Comes of Age. Trends Plant Sci..

[8] Cobb J.N., Juma R.U., Biswas P.S., Arbelaez J.D., Rutkoski J., Atlin G., Hagen T., Quinn M., Ng E.H. (2019). Enhancing the rate of genetic gain in public-sector plant breeding programs: Lessons from the breeder’s equation. Theor. Appl. Genet..

[9] Brim C.A. (1966). A Modified Pedigree Method of Selection in Soybeans 1. Crop Sci..

[10] Goulden C.H. (1939). Problems in Plant Selection.

[11] Borlaug N., Finlay K.W., Shephard K.W. (1968). Wheat Breeding and Its Impact on World Food Supply.

[12] Ghosh S., Watson A., Gonzalez-Navarro O.E., Ramirez-Gonzalez R.H., Yanes L., Mendoza-Suárez M., Simmonds J., Wells R., Rayner T., Green P. (2018). Speed breeding in growth chambers and glasshouses for crop breeding and model plant research. Nat. Protoc..

[13] Mobini S.H., Lulsdorf M., Warkentin T.D., Vandenberg A. (2016). Low red: Far-red light ratio causes faster in vitro flowering in lentil. Can. J. Plant Sci..

[14] Croser J.S., Pazos-Navarro M., Bennett R.G., Tschirren S., Edwards K., Erskine W., Creasy R., Ribalta F.M. (2016). Time to flowering of temperate pulses in vivo and generation turnover in vivo–in vitro of narrow-leaf lupin accelerated by low red to far-red ratio and high intensity in the far-red region. Plant Cell Tissue Organ Cult..

[15] Hickey L.T., Germán S.E., Pereyra S.A., Diaz J.E., Ziems L.A., Fowler R.A., Platz G.J., Franckowiak J.D., Dieters M.J. (2017). Speed breeding for multiple disease resistance in barley. Euphytica.

[16] Cazzola F., Bermejo C.J., Guindon M.F., Cointry E. (2020). Speed breeding in pea (*Pisum sativum* L.), an efficient and simple system to accelerate breeding programs. Euphytica.

[17] Jähne F., Hahn V., Würschum T., Leiser W.L. (2020). Speed breeding short-day crops by LED-controlled light schemes. Theor. Appl. Genet..

[18] Pfeiffer N.E. (1926). Microchemical and morphological studies of effect of light on plants. Bot. Gaz..

[19] Wheeler R.M. (2008). A historical background of plant lighting: An introduction to the workshop. Hortic. Sci..

[20] Sysoeva M.I., Markovskaya E.F., Shibaeva T.G. (2010). Plants under Continuous Light: A Review. Plant Stress.

[21] Pazos-Navarro M., Castello M., Bennett R.G., Nichols P., Croser J. (2017). In vitro-assisted single-seed descent for breeding-cycle compression in subterranean clover (*Trifolium subterraneum* L.). Crop Pasture Sci..

[22] Ribalta F.M., Pazos-Navarro M., Nelson K., Edwards K., Ross J.J., Bennett R., Munday C., Erskine W., Ochatt S.J., Croser J. (2017). Precocious floral initiation and identification of exact timing of embryo physiological maturity facilitate germination of immature seeds to truncate the lifecycle of pea. Plant Growth Regul..

[23] Ballare C.L., Scopel A.L., Stapleton A.E., Yanovsky M.J. (1996). Solar Ultraviolet-B Radiation Affects Seedling Emergence, DNA Integrity, Plant Morphology, Growth Rate, and Attractiveness to Herbivore Insects in Datura ferox. Plant Physiol..

[24] Singh D., Basu C., Meinhardt-Wollweber M., Roth B. (2015). LEDs for energy efficient greenhouse lighting. Renew. Sustain. Energy Rev..

[25] Christopher J., Richard C., Chenu K., Christopher M., Borrell A., Hickey L. (2015). Integrating Rapid Phenotyping and Speed Breeding to Improve Stay-Green and Root Adaptation of Wheat in Changing, Water-Limited, Australian Environments. Procedia Environ. Sci..

[26] Liu H., Zwer P., Wang H., Liu C., Lu Z., Wang Y., Yan G. (2016). A fast generation cycling system for oat and triticale breeding. Plant Breed..

[27] Mukade K. (1974). New Procedures for Accelerating Generation Advancement in Wheat Breeding. JARQ.

[28] Mobini S.H., Lulsdorf M., Warkentin T., Vandenberg A. (2014). Plant growth regulators improve in vitro flowering and rapid generation advancement in lentil and faba bean. Vitr. Cell. Dev. Biol.-Plant.

[29] Watson A., Ghosh S., Williams M.J., Cuddy W.S., Simmonds J., Rey M.-D., Hatta M.A.M., Hinchliffe A., Steed A., Reynolds D. (2018). Speed breeding is a powerful tool to accelerate crop research and breeding. Nat. Plants.

[30] Atieno J., Li Y., Langridge P., Dowling K., Brien C., Berger B., Varshney R., Sutton T. (2017). Exploring genetic variation for salinity tolerance in chickpea using image-based phenotyping. Sci. Rep..

[31] Ochatt S.J., Sangwan R.S., Marget P., Assoumou Ndong Y., Rancillac M., Perney P. (2002). New Approaches towards the Shortening of Generation Cycles for Faster Breeding of Protein Legumes. Plant Breed..

[32] Mobini S.H., Warkentin T.D. (2016). A simple and efficient method of in vivo rapid generation technology in pea (*Pisum sativum* L.). Vitr. Cell. Dev. Biol.-Plant.

[33] Rana M.M., Takamatsu T., Baslam M., Kaneko K., Itoh K., Harada N., Sugiyama T., Ohnishi T., Kinoshita T., Takagi H. (2019). Salt Tolerance Improvement in Rice through Efficient SNP Marker-Assisted Selection Coupled with Speed-Breeding. Int. J. Mol. Sci..

[34] Collard B.C.Y., Beredo J.C., Lenaerts B., Mendoza R., Santelices R., Lopena V., Verdeprado H., Raghavan C., Gregorio G.B., Vial L. (2017). Revisiting rice breeding methods—Evaluating the use of rapid generation advance (RGA) for routine rice breeding. Plant Prod. Sci..

[35] Forster B.P. (2014). Accelerated plant breeding. CAB Rev..

[36] Nagatoshi Y., Fujita Y. (2019). Accelerating Soybean Breeding in a CO_2_-Supplemented Growth Chamber. Plant Cell Physiol..

[37] Saxena K., Saxena R.K., Varshney R.K. (2017). Use of immature seed germination and single seed descent for rapid genetic gains in pigeonpea. Plant Breed..

[38] O'Connor D.J., Wright G.C., Dieters M.J., George D.L., Hunter M.N., Tatnell J.R., Fleischfresser D.B. (2013). Development and Application of Speed Breeding Technologies in a Commercial Peanut Breeding Program. Peanut Sci..

[39] Stetter M.G., Zeitler L., Steinhaus A., Kroener K., Biljecki M., Schmid K.J. (2016). Crossing Methods and Cultivation Conditions for Rapid Production of Segregating Populations in Three Grain Amaranth Species. Front. Plant Sci..

[40] Croser J., Mao D., Dron N., Michelmore S., McMurray L., Preston C., Bruce D., Ogbonnaya F.C., Ribalta F.M., Hayes J. (2021). Evidence for the Application of Emerging Technologies to Accelerate Crop Improvement—A Collaborative Pipeline to Introgress Herbicide Tolerance Into Chickpea. Front. Plant Sci..

[41] Lulsdorf M.M., Banniza S. (2018). Rapid generation cycling of an F2 population derived from a cross between *Lens culinaris* Medik. and *Lens ervoides* (Brign.) Grande after aphanomyces root rot selection. Plant Breed..

[42] Gosal S.S., Pathak D., Wani S.H., Vij S., Pathak M., Gosal S.S., Wani S.H. (2020). Accelerated Breeding of Plants: Methods and Applications. Accelerated Plant Breeding, Volume 1.

[43] Khoo K.H.P., Sheedy J.G., Taylor J.D., Croser J.S., Hayes J.E., Sutton T., Thompson J.P., Mather D.E. (2021). A QTL on the Ca7 chromosome of chickpea affects resistance to the root-lesion nematode *Pratylenchus thornei*. Mol. Breed..

[44] Dadu R.H.R., Bar I., Ford R., Sambasivam P., Croser J., Ribalta F., Kaur S., Sudheesh S., Gupta D. (2021). *Lens orientalis* Contributes Quantitative Trait Loci and Candidate Genes Associated with Ascochyta Blight Resistance in Lentil. Front. Plant Sci..

[45] Taylor C.M., Garg G., Berger J.D., Ribalta F.M., Croser J.S., Singh K.B., Cowling W.A., Kamphuis L.G., Nelson M.N. (2021). A Trimethylguanosine Synthase1-like (TGS1) homologue is implicated in vernalisation and flowering time control. Theor. Appl. Genet..

[46] Zaman S.U., Malik A.I., Kaur P., Ribalta F.M., Erskine W. (2019). Waterlogging Tolerance at Germination in Field Pea: Variability, Genetic Control, and Indirect Selection. Front. Plant Sci..

[47] Yao Y., Zhang P., Liu H., Lu Z., Yan G. (2017). A fully in vitro protocol towards large scale production of recombinant inbred lines in wheat (*Triticum aestivum* L.). Plant Cell Tissue Organ Cult..

[48] Ferrie A.M.R. (2007). Doubled haploid production in nutraceutical species: A review. Euphytica.

[49] Ortiz R., Trethowan R., Ferrara G.O., Iwanaga M., Dodds J.H., Crouch J.H., Crossa J., Braun H.-J. (2007). High yield potential, shuttle breeding, genetic diversity, and a new international wheat improvement strategy. Euphytica.

[50] Aldwinckle H.S. (1975). Flowering of apple seedlings 16–20 months after germination. Hortic. Sci..

[51] Van Nocker S., Gardiner S.E. (2014). Breeding better cultivars, faster: Applications of new technologies for the rapid deployment of superior horticultural tree crops. Hortic. Res..

[52] De Pauw R.M., Clarke J.M. (1976). Acceleration of generation advancement in spring wheat. Euphytica.

[53] Robertson L.D., Curtis B.C. (1967). Germination of Immature Kernels of Winter Wheat. Crop Sci..

[54] Tanaka J., Hayashi T., Iwata H. (2016). A practical, rapid generation-advancement system for rice breeding using simplified biotron breeding system. Breed. Sci..

[55] Ohnishi T., Yoshino M., Yamakawa H., Kinoshita T. (2011). The Biotron Breeding System: A Rapid and Reliable Procedure for Genetic Studies and Breeding in Rice. Plant Cell Physiol..

[56] Alahmad S., Dinglasan E., Leung K.M., Riaz A., Derbal N., Voss-Fels K.P., Able J.A., Bassi F.M., Christopher J., Hickey L.T. (2018). Speed breeding for multiple quantitative traits in durum wheat. Plant Methods.

[57] Zhang Z., Wei W., Zhu H., Challa G.S., Bi C., Trick H.N., Li W. (2015). W3 Is a New Wax Locus That Is Essential for Biosynthesis of β-Diketone, Development of Glaucousness, and Reduction of Cuticle Permeability in Common Wheat. PLoS ONE.

[58] Rizal G., Karki S., Alcasid M., Montecillo F., Acebron K., Larazo N., Garcia R., Slamet-Loedin I.H., Quick W.P. (2014). Shortening the Breeding Cycle of Sorghum, a Model Crop for Research. Crop Sci..

[59] Burris J.S. (1973). Effect of Seed Maturation and Plant Population on Soybean Seed Quality. Agron. J..

[60] Roumet P., Morin F. (1997). Germination of Immature Soybean Seeds to Shorten Reproductive Cycle Duration. Crop Sci..

[61] Dagustu N., Bayram G., Sincik M., Bayraktaroglu M. (2012). The Short Breeding Cycle Protocol Effective on Diverse Genotypes of Sunflower (*Helianthus annuus* L.). Turkish J. Field Crop..

[62] Murashige T., Skoog F. (1962). A Revised Medium for Rapid Growth and Bio Assays with Tobacco Tissue Cultures. Physiol. Plant..

[63] Espósito M.A., Almirón P., Gatti I., Cravero V.P., Anido F.S.L., Cointry E.L. (2012). Methodology A rapid method to increase the number of F1 plants in pea (*Pisum sativum*) breeding programs. Genet. Mol. Res..

[64] Samineni S., Sen M., Sajja S.B., Gaur P.M. (2020). Rapid generation advance (RGA) in chickpea to produce up to seven generations per year and enable speed breeding. Crop J..

[65] Parmar S., Deshmukh D.B., Kumar R., Manohar S.S., Joshi P., Sharma V., Chaudhari S., Variath M.T., Gangurde S.S., Bohar R. (2021). Single Seed-Based High-Throughput Genotyping and Rapid Generation Advancement for Accelerated Groundnut Genetics and Breeding Research. Agronomy.

[66] Baier K., Maynard C., Powell W.A. (2012). Early Flowering in Chestnut Species Induced under High Intensity, High Dose Light in Growth Chambers. J. Am. Chestnut Found..

[67] Flachowsky H., Le Roux P.-M., Peil A., Patocchi A., Richter K., Hanke M.-V. (2011). Application of a high-speed breeding technology to apple (*Malus* × *domestica*) based on transgenic early flowering plants and marker-assisted selection. New Phytol..

[68] Vira B., Wildburger C., Mansourian S., International Union of Forestry Research Organizations (2015). Forests, Trees and Landscapes for Food Security and Nutrition: A Global Assessment Report.

[69] Souza L.S., Diniz R.P., Neves R., Alves A.A.C., de Oliveira E.J. (2018). Grafting as a strategy to increase flowering of cassava. Sci. Hortic..

[70] Demers D.-A., Dorais M., Wien C.H., Gosselin A. (1998). Effects of supplemental light duration on greenhouse tomato (*Lycopersicon esculentum* Mill.) plants and fruit yields. Sci. Hortic..

[71] Bhattaraj S.P., de la Pena R.C., Midmore D.J., Palchamy K. (2009). In vitro culture of immature seed for rapid generation advancement in tomato. Euphytica.

[72] Manzur J., Oliva-Alarcón M., Rodríguez-Burruezo A. (2014). In vitro germination of immature embryos for accelerating generation advancement in peppers (*Capsicum annuum* L.). Sci. Hortic..

[73] Geboloğlu N., Bozmaz S., Aydin M., Cakmak P. (2011). The role of growth regulators, embryo age and genotypes on immature embryo germination and rapid generation advancement in tomato (*Lycopersicon esculentum* Mill.). Afr. J. Biotechnol..

[74] Borovsky Y., Mohan V., Shabtai S., Paran I. (2020). CaFT-LIKE is a flowering promoter in pepper and functions as florigen in tomato. Plant Sci..

[75] Velez-Ramirez A.I., Van Ieperen W., Vreugdenhil D., Van Poppel P.M.J.A., Heuvelink E., Millenaar F.F. (2014). A single locus confers tolerance to continuous light and allows substantial yield increase in tomato. Nat. Commun..

[76] Yang H., Wu J.-J., Tang T., Liu K.-D., Dai C. (2017). CRISPR/Cas9-mediated genome editing efficiently creates specific mutations at multiple loci using one sgRNA in *Brassica napus*. Sci. Rep..

[77] Murovec J., Guček K., Bohanec B., Avbelj M., Jerala R. (2018). DNA-Free Genome Editing of *Brassica oleracea* and *B. rapa* Protoplasts Using CRISPR-Cas9 Ribonucleoprotein Complexes. Front. Plant Sci..

[78] Bao A., Zhang C., Huang Y., Chen H., Zhou X., Cao D. (2020). Genome editing technology and application in soybean improvement. Oil Crop Sci..

[79] Angeles-Shim R.B., Reyes V.P., del Valle M.M., Lapis R.S., Shim J., Sunohara H., Jena K.K., Ashikari M., Doi K. (2020). Marker-Assisted Introgression of Quantitative Resistance Gene pi21 Confers Broad Spectrum Resistance to Rice Blast. Rice Sci..

[80] Voss-Fels K.P., Herzog E., Dreisigacker S., Sukurmaran S., Watson A., Frisch M., Hayes B.J., Hickey L.T., Miedaner T., Korzun V. (2018). Speed GS to accelerate genetic gain in spring wheat. Applications of Genetic and Genomic Research in Cereals.

[81] Watson A., Hickey L.T., Christopher J., Rutkoski J., Poland J., Hayes B.J. (2019). Multivariate Genomic Selection and Potential of Rapid Indirect Selection with Speed Breeding in Spring Wheat. Crop Sci..

[82] Al Tamimi N., Brien C., Oakey H., Berger B., Saade S., Ho Y.S., Schmöckel S.M., Tester M., Negrao S. (2016). Salinity tolerance loci revealed in rice using high-throughput non-invasive phenotyping. Nat. Commun..

[83] Richard C., Hickey L., Fletcher S., Chenu K., Borrell A., Christopher J. (2015). High-throughput Phenotyping of Wheat Seminal Root Traits in a Breeding Context. Procedia Environ. Sci..

[84] Walter J., Edwards J., Cai J., McDonald G., Miklavcic S., Kuchel H. (2019). High-Throughput Field Imaging and Basic Image Analysis in a Wheat Breeding Programme. Front. Plant Sci..

[85] Phyu P., Islam M.R., Cruz P.C.S., Collard B.C.Y., Kato Y. (2020). Use of NDVI for indirect selection of high yield in tropical rice breeding. Euphytica.

[86] Samantara K., Reyes V.P., Agrawal N., Mohapatra S.R., Jena K.K. (2021). Advances and trends on the utilization of multi-parent advanced generation intercross (MAGIC) for crop improvement. Euphytica.

[87] Kitony J.K., Sunohara H., Tasaki M., Mori J.-I., Shimazu A., Reyes V., Yasui H., Yamagata Y., Yoshimura A., Yamasaki M. (2021). Development of an *Aus*-Derived Nested Association Mapping (*Aus*-NAM) Population in Rice. Plants.

[88] Wanga M.A., Shimelis H., Mark J.M., Laing D. (2021). Opportunities and challenges of speed breeding: A review. Plant Breed..

[89] Barrios P.M.G., Bhatta M., Halley M., Sandro P., Gutiérrez L. (2020). Speed breeding and early panicle harvest accelerates oat (*Avena sativa* L.) breeding cycles. Crop Sci..

[90] Sharma A., Jones J.B., White F.F. (2019). Recent advances in developing disease resistance in plants. F1000Research.

[91] Bennett R., Ribalta F.M., Pazos-Navarro M., Leonforte A., Croser J.S. (2017). Discrimination of boron tolerance in *Pisum sativum* L. genotypes using a rapid, high-throughput hydroponic screen and precociously germinated seed grown under far-red enriched light. Plant Methods.

[92] Varshney R.K., Bohra A., Roorkiwal M., Barmukh R., Cowling W.A., Chitikineni A., Lam H.-M., Hickey L.T., Croser J.S., Bayer P.E. (2021). Fast-forward breeding for a food-secure world. Trends Genet..

